# Relating corynebacterial cell envelope layers to its biochemical and physical properties

**DOI:** 10.1016/j.tcsw.2025.100162

**Published:** 2025-11-16

**Authors:** Mamata Modak, Uchenna Watson Waturuocha, Deepak Kumar Saini, Apoorva Bhatt, Gurdyal S. Besra

**Affiliations:** a School of Biosciences and Institute of Microbiology and Infection, University of Birmingham, Edgbaston, Birmingham B15 2TT, United Kingdom; b Department of Molecular Reproduction, Development and Genetics, Indian Institute of Science, Bangalore, India

**Keywords:** Corynebacterium, Mycobacterium, Cell wall, Mutants, Biophysical

## Abstract

The cell wall plays a central role in maintaining bacterial shape and structural stability. In the *Corynebacteriaceae* family, which includes *Corynebacterium*, *Mycobacterium*, *Nocardia*, and *Rhodococcus*, the envelope has a distinctive organization: peptidoglycan is covalently attached to arabinogalactan, which is subsequently enveloped by an outer layer of mycolic acids. In this study, we used *Corynebacterium glutamicum* as a model to examine how disruptions in different layers of the cell envelope, specifically the arabinogalactan and mycolic acid layers, influence both biochemical and biophysical properties. We assessed surface hydrophobicity, antibiotic sensitivity, and lipid composition, and complemented these assays with atomic force microscopy to examine structural changes. Our analysis of multiple cell wall mutants suggests that a division of labour occurs among the layers of the corynebacterial envelope, where each layer contributes to distinct, but complementary functions to overall cell wall physiology.

## 1 Introduction

**Fig. 1:**
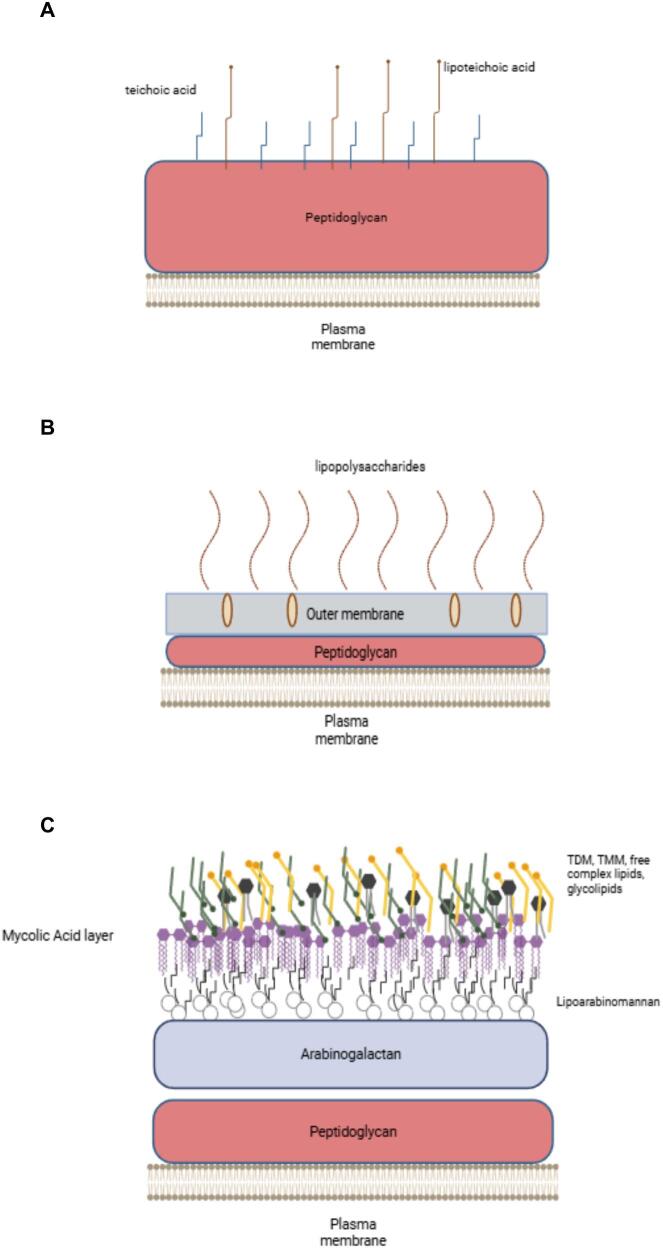
Structure organization of cell walls. A) Gram-positive bacteria B) Gram-negative bacteria C) Corynebacterial/Mycobacterial cell wall framework.

For most bacteria, the maintenance of cell shape and structural integrity relies on the intricate architecture of their cell wall, which also provides defence against harsh environments, mechanical resistance, and facilitates the transport of solutes and proteins, as well as adherence to receptors (Chiaradia et al., 2017). Bacteria are classified as Gram-positive or Gram-negative based on their cell wall structure. Gram-positive bacteria have a thick peptidoglycan layer and lack an outer membrane containing lipopolysaccharides (Fig. 1A). In contrast, Gram-negative bacteria possess a thinner peptidoglycan layer encased by an outer membrane rich in lipopolysaccharides (Fig. 1B) (Pasquina-Lemonche et al., 2020). This fundamental difference in cell wall composition has significant implications for their biology and antibiotic sensitivity (Silhavy et al., 2010).

Mycobacterial cell walls exhibit a highly complex structure compared to Gram-positive and Gram-negative bacteria (Fig. 1C). A defining feature is the mycolyl-arabinogalactan-peptidoglycan complex (mAGP), composed of mycolic acids covalently linked to arabinogalactan, which in turn is attached to a cross-linked peptidoglycan network that forms the core of the cell wall (Brennan and Nikaido, 1995; Brennan, 2003). Intercalated within this mycolate layer is an outer membrane that contains solvent-extractable lipids, such as glycolipids and inert waxes (Minnikin et al., 2015). Mycobacterial cell envelopes contain a significant amount of lipids, accounting for up to 40 % of their dry weight (Daffé and Draper, 1997). The lipid-rich cell wall of mycobacteria is responsible for their acid-fast characteristic. This distinct structure, especially the mycolic acids, forms a barrier that retains dye during staining, enabling mycobacteria to withstand decolorization by acid-alcohol solutions (Daffé and Marrakchi, 2019). The mAGP complex also plays a crucial role in the viability of *Mycobacterium tuberculosis* (Alderwick et al., 2015; Tran et al., 2019; Birhanu et al., 2019), contributing to the permeability barrier characteristic of mycobacteria. The biosynthesis of each component of the mycobacterial cell wall involves a sophisticated and intricate machinery, making it a challenging yet critical area of study. Additionally, the cell wall plays a significant role in antibiotic resistance (Brennan and Nikaido, 1995; Brennan, 2003). Research has also indicated that various cell wall components distinctly influence the pathogenesis of mycobacteria (Singh et al., 2017). This highlights the critical importance of studying the cell wall. Gaining insights into its structure and function can pave the way for developing more effective treatments and strategies.

Changes in the cell wall can alter a bacterium's physical and biochemical properties, enabling us to understand how these factors relate to the bacteria's survival and viability (Sun et al., 2022). The complex lipids in the cell wall of *M. tuberculosis* have a critical function in host interactions (Ghazaei, 2018). These lipids are organized into distinct layers: the inner leaflet consists of α-alkyl, β-hydroxy long-chain mycolic acids, while the outer leaflet contains non-covalently bound lipids and glycolipids. The lipids found, include trehalose monomycolates, trehalose dimycolates, phenolic glycolipids, phthiocerol dimycocerosates, poly-acylated trehaloses, glycerol and glucose monomycolates, sulfolipids, phosphatidylinositol mannosides, and mannose-capped lipoarabinomannans (Gago et al., 2018). Mycolic acids are a hallmark of the *M. tuberculosis* cell envelope, contributing to its structural integrity and biophysical properties. Their composition directly affects the flexibility of the envelope. Additionally, their strong hydrophobicity is a key determinant of virulence, facilitating host-pathogen interactions and resistance to environmental stress (Ghazaei, 2018).

Cell surface hydrophobicity is a critical property for bacteria, influencing adhesion (Zita and Hermansson, 1997), virulence, and the aerosol transmission of pathogenic species of mycobacteria (Jankute et al., 2017). The hydrophobic characteristics of microorganisms are also linked to their ability to form biofilms on abiotic surfaces (Gomes et al., 2013). Research indicates that the attachment of *Listeria monocytogenes* to PVC surfaces, and the subsequent biofilm formation, correlates with adherence to hexadecane (Takahashi et al., 2010). Given that hydrophobicity can alter infection dynamics and diminish the efficacy of antimicrobial therapies, it carries significant therapeutic implications (Gomez et al., 2013). Notably, studies have shown that pathogenic mycobacteria exhibit an enhanced evolutionary hydrophobicity (Jankute et al., 2017).

Morphological changes in mycobacteria can indicate several key aspects, including their growth phase and pathogenicity. In certain mycobacterial species, including *M. tuberculosis, M. avium,* and *M. abscessus*, a shift to the rough phenotype is linked to more severe infections compared to the smooth phenotype (Born et al., 2023; Leestemaker-Palmer et al., 2025). For example, mutations in the *mps1-mps2-gap* or *mmpL4b* gene clusters, which are involved in glycopeptidolipid synthesis and transport, can trigger the conversion from the smooth form to the rough form in *M. abscessus* (Chung et al., 2022). These changes in the cell surface can be studied using atomic force microscopy (AFM), which provides real-time, three-dimensional images of surface ultrastructure with molecular resolution and minimal sample preparation (Dufrêne, 2002). Force measurements will allow us to explore the physical properties of the material, including molecular interactions, surface hydrophobicity, surface charges, and mechanical properties (Huang et al., 2015). These measurements offer a novel perspective on microbial surface structure and function.

Understanding how modifications in the cell wall integrity hold important implications for addressing infections caused by *M. tuberculosis* and *M. abscessus*. These organisms are notoriously difficult to treat due to their highly impermeable cell envelopes and capacity to form resilient biofilms (Dokic et al., 2021; Chakraborty et al., 2021). By dissecting how cell wall composition shapes mechanical stability and surface hydrophobicity, studies in *C. glutamicum* can uncover critical vulnerabilities that may be exploited for more precise antibiotic targeting or rational combination therapies. In addition, correlating structural alterations with biofilm formation provides a framework for understanding bacterial persistence and for designing strategies to improve treatment outcomes in chronic infections.

*Corynebacterium* is a member of the Corynebacteriaceae family, which includes the genera *Mycobacteria*, *Nocardia*, and *Rhodococcus* (Zhi et al., 2017). *Corynebacterium* species possess complex cell wall architectures, featuring peptidoglycan covalently linked to an arabinogalactan meshwork, along with an outer layer of mycolic acids. The outermost layer comprises free polysaccharides, glycolipids, proteins, pili, and S-layer proteins (Burkovski, 2013). Both *Mycobacterium* and *Corynebacterium* species share the characteristic mAGP architecture, with homologous pathways for synthesising and assembling these layers. Key genes involved in arabinogalactan biosynthesis, such as the *emb* and *aft* are highly conserved (Alderwick et al., 2006; Alderwick et al., 2005). Despite differences in lipid complexity and the presence of additional virulence-associated lipids in pathogenic mycobacteria (Dover et al., 2004), a shared framework means that *C. glutamicum* provides a robust and genetically tractable model for dissecting the organization and function of the mAGP cell envelope.

**Fig. 2:**
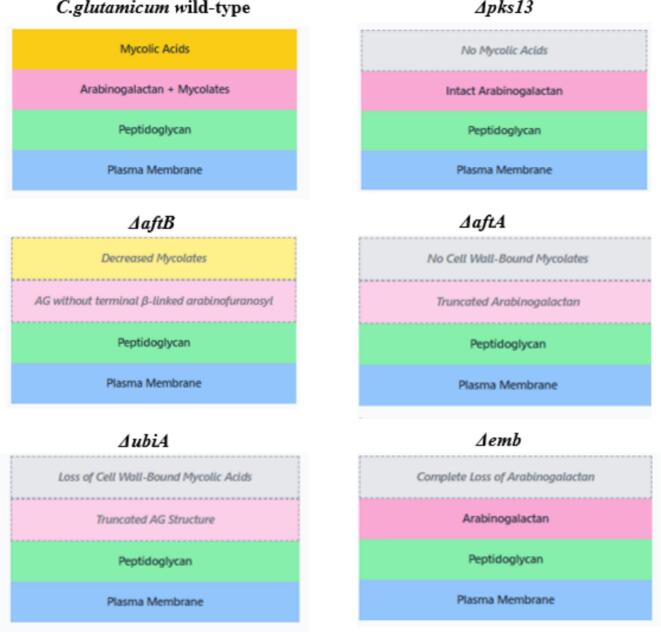
Cell envelope phenotypes of *C.glutamicum* wild-type and mutants.

We selected *C. glutamicum* as a model organism due to its resilience. In this study, we use defined mutants lacking different components of the cell wall (Fig. 2) to assess the role each layer plays in its biochemical (lipid profile, antibiotic susceptibility, hydrophobicity) and biophysical (elasticity) characteristics.

## 2 Materials and methods

### 2.1 Bacterial strains and culture conditions

The following *C. glutamicum* strains were used in this study (Table 1) and grown in BHIS media (37 g/Lbrain-heart infusion media, 91 g/L sorbitol) at 30 °C in shaking conditions. Δ*ubiA* strain is grown in Kan 25 μg/mL. BHIS agar plates with 100 μg/mL Congo red were used.

**Table 1:** *C. glutamicum* cell wall mutant strains used in this study.

Strains	Function of the gene and phenotype	Source
Δ*pks13*	Pks13 is involved in the final steps of mycolic acid synthesis	Gande et al., 2004
	No mycolic acids production, intact arabinogalactan (AG) present	
Δ*aftB*	AftB is responsible for the transfer of Araf residues from DPA to the arabinan domain.	Seidel et al., 2007
	Complete absence of terminal β-linked arabinofuranosyl residues, decreased cell-wall bound mycolates	
Δ*aftA*	AftA helps in addition of the first key Araf residues to the galactan domain of AG.	Alderwick et al., 2006
	Complete absence of arabinose resulting in a truncated cell wall structure, loss of cell wall bound mycolates	
Δ*emb*	Emb is involved in AG polymerization	Alderwick et al., 2005
	Truncated AG structure, loss of cell wall bound mycolic acids	
Δ*ubiA* (Kanamycin resistance)	UbiA is an enzyme involved in the biosynthesis of the sugar donor decaprenol phosphoarabinose (DPA)	Alderwick et al., 2005
	Complete loss of cell wall arabinan	

### 2.2 Extraction and analysis of ^14^C-labelled lipids

#### 2.2.1 Radioactive labelling

*C. glutamicum* wild-type and mutant strains were streaked onto BHIS agar plates and incubated at 30 °C for 24 to 48 h (for slow-growing mutants). Single colonies were inoculated into 5 mL BHIS broth and grown overnight at 30 °C with shaking (180 rpm). A 1 mL aliquot of the overnight culture was used to inoculate 10 mL BHIS broth for secondary culture. Cultures were grown at 30 °C with shaking until reaching an OD (600 nm) of approximately 0.4. At this density, cultures were labelled with 5 μCi/mL [^14^C]-acetate (Carbon-14 labelled acetate, used as a metabolic tracer). Cultures were incubated for an additional 24 to 48 h at 30 °C, before harvesting by centrifugation (4000 rpm, 10 min). Pellets were washed once with PBS and dried under a gentle nitrogen stream or in a vacuum concentrator.

#### 2.2.2 Extraction of total lipids

Lipid extraction followed standard protocols for *Corynebacteriales* cell envelopes (from Dobson et al., 1985; Minnikin et al., 1982). Densitometric analyses were performed using the ImageJ software. Results are expressed as the mean ± standard deviation of at least three independent experiments. Statistical significance was determined by Student's *t*-test (*p* < 0.01).

#### 2.2.3 Extraction and analysis of mycolic acid methyl esters (MAMEs) and fatty acid methyl esters (FAMEs)

This method follows established tetra-butyl ammonium hydroxide (TBAH)-based hydrolysis and methylation procedures (Butler et al., 1991; Garton et al., 2002). Thin-layer chromatography was performed using Silica Gel 60 F254 plates from Merck. For the total lipid analysis, chloroform:methanol: water (60:16:2, *v*/*v*/v), 20,000 cpm (counts per minute) of each strain, was used. For FAMEs and MAMEs, petroleum-ether (B.P. 60 °C–80 °C)/acetone (95:5, v/v) was used for normal phase TLCs. To expose the [^14^C]-labelled lipids, autoradiograms were created by exposing Kodak X-Omat AR film for four to five days.

### 2.3 Hexadecane partitioning assay

The hydrophobicity of *C. glutamicum* strains was assessed using a hexadecane partitioning assay adapted from Rosenberg et al. (1980). Log-phase cultures of the wild-type and mutants were harvested by centrifugation, washed three times with PUM- Phosphate Urea Magnesium buffer, (22.2 mM potassium phosphate, 22.2 mM magnesium sulfate, and 100 mM urea; pH 7.1), and resuspended to an OD (600 nm) of 0.7. A 3 mL aliquot of the cell suspension was transferred to a clean glass tube, after which 2.4 mL of hexadecane was added. The mixture was briefly vortexed to allow interaction between the cells and the hydrocarbon and then incubated for 8 min at 37 °C, followed by 15 min at 22 °C to permit phase separation. The optical density of the aqueous phase was measured at 600 nm and expressed as a percentage relative to the OD (600 nm) of the bacterial suspension in PUM buffer alone, which was used to calculate the hydrophobicity index. Results are expressed as the mean ± standard deviation of at least three independent experiments. Statistical significance was determined by Student's *t*-test (*p* < 0.01).

### 2.4 Minimum inhibitory concentration

To compare antibiotic susceptibility between wild-type and mutant strains of *C. glutamicum*, we determined the MIC_90_ of rifampicin and streptomycin using a resazurin-based microdilution assay (Palomino et al., 2002). Rifampicin and streptomycin stock solutions were prepared in dimethyl sulfoxide (DMSO) and sterile water, respectively. Overnight cultures grown in BHIS at 30 °C were diluted to an OD (600 nm) of 0.005 in fresh BHIS medium, and 20 μL was added to each well to achieve a starting OD (600 nm) of 0.0005 and added to 96-well plates containing a two-fold dilution series of each antibiotic. Plates were incubated statically at 30 °C for 24 h. Following incubation, 20 μL of 0.02 % resazurin solution (filter sterilised in distilled water) was added to each well, and plates were incubated for an additional 1- 2 h at 30 °C. Fluorescence was measured using a microplate reader at excitation and emission wavelengths of 560 nm and 590 nm, respectively. Percent survival was calculated relative to untreated controls, and MIC_90_ were defined as the lowest antibiotic concentration at which survival dropped below 10 %. Results are expressed as the mean ± standard deviation of at least three independent experiments. Statistical significance was determined by Student's *t*-test (*p* < 0.01).

### 2.5 Atomic force microscopy

Single colonies of each bacterial culture were inoculated into BHIS broth and grown at 30 °C with shaking (180 rpm), cultures were grown until reaching mid-log phase. Cells were collected by centrifugation at 4500 rpm for 5 min and washed three times with sterile Milli-Q water to remove residual media components. The OD at 600 nm was adjusted to 0.1, and 100 μL of the suspension was placed onto poly-l-lysine–coated coverslips to facilitate surface adhesion. Samples were allowed to air-dry in a laminar flow hood at room temperature for 1 h. A wash step with MilliQ water was performed to remove loosely attached bacteria, after which the coverslips were air-dried for an additional hour. The dried coverslips were mounted onto magnetic stubs using double-sided carbon tape before AFM analysis.

AFM measurements were conducted using a NX-10 Atomic Force Microscope (Park Systems, South Korea) operating in non-contact mode. A high-stiffness silicon cantilever with a pyramidal tip (tip radius < 10 μm; width: 35 μm; length: 125 μm; thickness: 4.5 μm) was used, oscillating at a resonance frequency of 300 kHz. All imaging was performed under ambient conditions (Waturuocha et al., 2021).

## 3 Results

### 3.1 Comparative lipid analysis of *C. glutamicum* cell wall mutant strains

To examine the effect of disrupting key genes involved in the biosynthesis and assembly of the cell envelope, we carried out a detailed analysis of lipid profiles of the *C. glutamicum* cell wall mutants. Specifically, we assessed the abundance of trehalose monocorynomycolates (TMCM) and trehalose dicorynomycolates (TDCM), which serve as major mycolic acid–containing glycolipids in the mycomembrane.

**Fig. 3:**
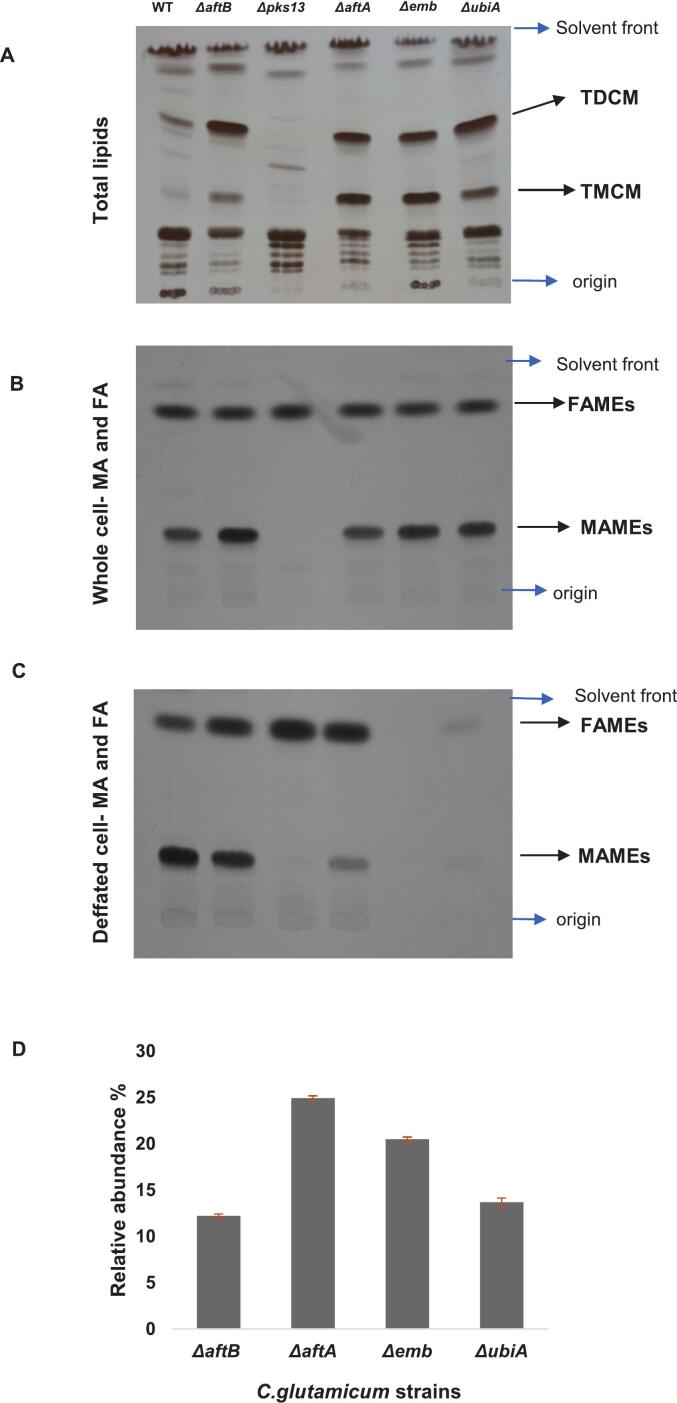
**1D TLC autoradiography** of [^14^C]-acetic acid-labelled lipids from Wild type (WT) and mutants (Δ*aftB,* Δ*pks13,* Δ*aftA,* Δ*emb,* Δ*ubiA)*. A) Total free lipid extracts B) Whole cell FAMES and MAMES extracts C) FAMES and MAMES extracts of defatted cells. D) Densitometry of TMCM represented relative to the wild-type.

Compared to the wild-type strain, the Δ*aftB*, Δ*ubiA*, Δ*aftA*, and Δ*emb* mutants all displayed increased accumulation of both TMCM and TDCM (Fig. 3A). This accumulation likely reflects impaired incorporation of mycolic acids into the arabinogalactan–peptidoglycan complex or defective transport of these glycolipids across the membrane. Interestingly, the degree of TMCM accumulation varied among the mutants: while Δ*aftA* and Δ*emb* showed robust increases, both Δ*aftB* and Δ*ubiA* displayed comparatively lower levels of TMCM (Fig. 3D). These differences may reflect distinct steps in the biosynthetic pathway affected by each mutation, such as precursor synthesis (Δ*ubiA*), polymer modification (Δ*aftB*), or structural integration into the cell wall matrix.

In contrast to the above mutants, the Δ*pks13* strain showed a complete absence of both TMCM and TDCM, aligning with the essential role of Pks13 in catalyzing the final condensation step in mycolic acid biosynthesis. Remarkably, this strain also exhibited accumulation of an unidentified lipid species not present in the wild type or other mutants. This may represent a biosynthetic intermediate or shunt metabolite, suggesting that in the absence of mycolate production, metabolic flux is redirected toward alternate lipid biosynthetic pathways.

To directly assess the presence of mycolic acids and potential changes in fatty acid content, we extracted and analyzed MAMEs and FAMEs from both whole cells and defatted cell preparations. In whole-cell extracts (Fig. 3B), consistent with glycolipid profiling, MAMEs were completely absent in the Δ*pks13* mutant, confirming the loss of mycolic acid synthesis; the other mutants, Δ*aftB*, Δ*ubiA*, Δ*aftA*, and Δ*emb* displayed MAME profiles largely similar to the wild type. However, after defatting (Fig. 3C), substantial differences emerged: both Δ*pks13* and Δ*emb* mutants showed a complete loss of extractable MAMEs, suggesting that either mycolic acids are not synthesized (Δ*pks13*) or are present in non-covalently bound pools that are lost during delipidation (Δ*emb*). Notably, Δ*aftA* and Δ*ubiA* retained reduced but detectable MAMEs, further indicating partial defects in mycolate attachment or envelope retention.

FAMEs analysis revealed additional insights into the compensatory lipid metabolism in these mutants. Elevated levels of fatty acid methyl esters were detected in the Δ*pks13*, Δ*aftA*, and Δ*aftB* strains, which may reflect the accumulation of unutilized fatty acid precursors or compensatory remodelling of membrane phospholipids in response to disrupted mycolic acid pathways. Conversely, Δ*emb* lacked detectable FAMEs, suggesting a broader disruption in lipid biosynthesis or extraction efficiency linked to its role in arabinan polymerization. The Δ*ubiA* mutant exhibited markedly reduced FAME content, further supporting the hypothesis that perturbation in the early steps of arabinogalactan assembly significantly influences global lipid homeostasis.

Together, these results demonstrate that genetic disruption of key cell wall biosynthetic genes leads to distinct and predictable alterations in both mycolic acid and fatty acid composition. The accumulation of mycolate-containing glycolipids in several mutants' points to a bottleneck in the final steps of envelope assembly, while the complete loss of mycolates in Δ*pks13* underscores the central role of this gene in initiating the mycolate arm of lipid biosynthesis. These findings further support the view that the composition and organization of the mycomembrane are tightly regulated and intimately linked to the coordinated activity of multiple biosynthetic modules across the envelope.

### 3.2 Impact of cell envelope perturbation on hydrophobicity

To investigate alterations in cell surface hydrophobicity associated with disruption of the mycolic acid biosynthesis pathway, we employed the hexadecane partitioning assay as originally described by Rosenberg et al. (1980). This assay is based on the differential affinity of bacterial cells for an aqueous versus a hydrophobic phase (n-hexadecane), following vigorous mixing. The extent to which cells partition into the hydrocarbon phase serves as a proxy for cell surface hydrophobicity and reflects compositional and structural changes in the outer cell envelope.

**Fig. 4:**
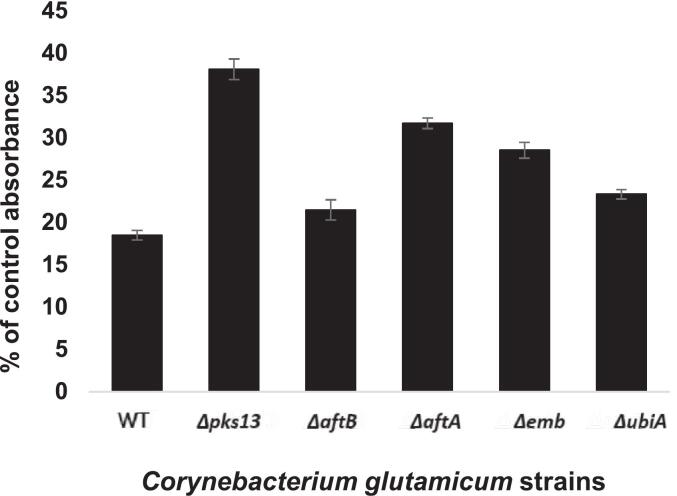
**Hexadecane partitioning of live cells of WT and cell wall mutants**. Results are expressed as mean ± standard deviation of at least three independent experiments. Statistical significance was determined by Student's *t*-test (*p* < 0.01).

In this study, wild-type *C. glutamicum* exhibited the highest affinity for the hydrophobic phase, consistent with its known mycolic acid-rich outer membrane (Fig. 4). In contrast, all tested cell wall mutants showed reduced partitioning into the hexadecane phase, indicating a decrease in surface hydrophobicity. Among the mutants, the ∆*pks13* strain showed the most pronounced reduction, suggesting that complete loss of mycolic acid synthesis severely compromises the hydrophobic character of the cell surface. The ∆*aftA*, ∆*emb*, and ∆*ubiA* mutants also displayed a significant decrease in hydrophobicity relative to the wild type, consistent with their roles in the biosynthesis and assembly of the mycoloyl-arabinogalactan–peptidoglycan complex.

Interestingly, the ∆*aftB* mutant exhibited the least reduction in hydrophobicity among the mutants, which may be attributed to its partial retention of mycolic acids, as observed in previous lipid profiling studies. This suggests that even a partial complement of mycolic acids is sufficient to confer a degree of hydrophobicity, although not to the extent seen in the wild type. Taken together, these results underscore the critical role of mycolic acids in modulating cell surface properties and highlight the contribution of specific biosynthetic genes in maintaining the hydrophobic nature of the corynebacterial envelope.

### 3.3 Antibiotic susceptibility

To investigate the functional consequences of disrupted cell envelope integrity, we examined the antibiotic susceptibility profiles of *C. glutamicum* wild-type and the mutants. Two antibiotics with distinct mechanisms of action and physicochemical properties were selected for this study: rifampicin and streptomycin. Rifampicin is a lipophilic antibiotic that must penetrate the hydrophobic membrane to inhibit bacterial RNA polymerase (Arriaga-Guerrero et al., 2020). It is widely used to probe envelope permeability in mycolata, as its activity is known to be influenced by cell wall architecture and mycolic acid content. In contrast, streptomycin, a hydrophilic aminoglycoside, targets the 30S subunit of the ribosome and enters cells via energy-dependent pathways rather than passive diffusion across the cell envelope (Delcour, 2009). As such, streptomycin serves as a useful control to distinguish between changes in antibiotic uptake driven by altered envelope permeability versus broader physiological effects.

No significant differences in streptomycin susceptibility were observed across the mutant panel (Table 2), indicating that loss of mycolic acids or other envelope components does not broadly affect the activity of hydrophilic antibiotics that rely on active transport mechanisms. However, a distinct pattern emerged with rifampicin. The wild-type strain, possessing an intact and fully mycolylated cell envelope, exhibited a rifampicin MIC_90_ of 12 ng/mL. In contrast, strains with compromised mycolic acid content displayed increased rifampicin sensitivity. The Δ*aftB* mutant, which retains a partial pool of mycolic acids, showed a moderate increase in susceptibility with an MIC_90_ of 7 ng/mL. This suggests that even partial disruption of mycolic acid incorporation is sufficient to reduce the barrier function of the cell wall, enhancing rifampicin penetration.

**Table 2:** MIC_90_ of *C. glutamicum* strains.

Strain	Rifampicin (ng/ml)	Streptomycin (μg/ml)
*C.glutamicum* WT	12	5
Δ*aftB*	7	5
Δ*aftA*	4	5
Δ*pks13*	4	5
Δ*emb*	4	5
Δ*ubiA*	4	5

Strikingly, the Δ*pks13*, Δ*aftA*, Δ*emb*, and Δ*ubiA* mutants exhibited a pronounced decrease in MIC_90_ values for rifampicin, indicative of heightened susceptibility. These strains are characterized by a near-complete loss of covalently bound mycolic acids, as confirmed by lipid and methyl ester profiling. The heightened rifampicin sensitivity in these mutants strongly supports the role of mycolic acids in limiting the diffusion of lipophilic antibiotics through the cell envelope. The correlation between reduced mycolate abundance and increased drug susceptibility suggests that the mycomembrane serves as a key permeability barrier, and that its disruption can profoundly affect antibiotic access to intracellular targets.

These results highlight the protective function of the mycolic acid–rich envelope in antibiotic resistance, particularly against lipophilic compounds such as rifampicin. Moreover, they reinforce the concept that targeting mycolic acid biosynthesis can serve as an effective strategy to enhance the efficacy of existing antibiotics by weakening the permeability barrier. This approach could be especially valuable in the context of multidrug-resistant corynebacteria or related actinobacteria.

### 3.4 Atomic force microscopy

To understand how different components of the corynebacterial cell envelope contribute to its mechanical properties, we measured the stiffness of wild-type and mutant strains using atomic force microscopy (AFM)-based nanoindentation. The stiffness of the membrane is a direct measure of its elasticity/rigidity and permeability (Auer and Weibel, 2017). The stiffness was assessed by calculating Young's modulus, which is a measure of how the membrane of the cell responds to deformation under applied force.

**Fig. 5:**
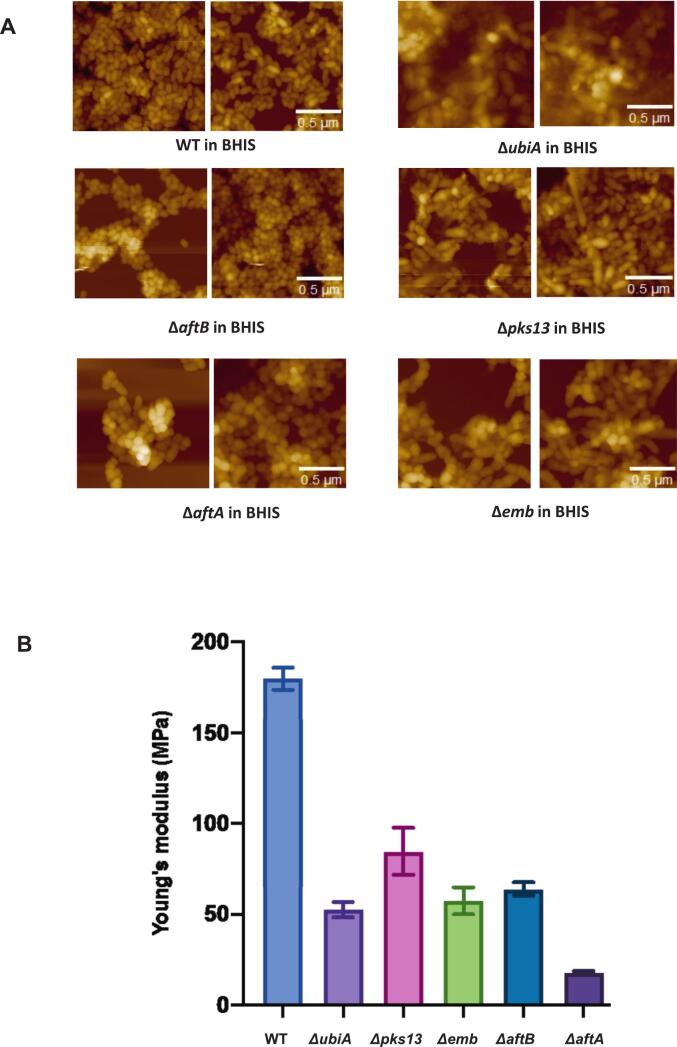
**Young's modulus measurements (MPa) of WT and cell wall mutants**. A) Representative images of 2 different ROIs of WT and mutant strains used for AFM measurements. B) Young's modulus (MPa) measurements of WT and mutant strains. Results are expressed as mean ± standard deviation of at least three independent experiments. Statistical significance was determined by Student's *t*-test (p < 0.01).

The wild-type cells appeared as smooth, rounded rods forming dense aggregates, consistent with the typical morphology of *C. glutamicum* under these conditions (Fig. 5A). Subtle variations in surface texture could be observed in the AG mutants, such as slightly rougher or more irregular surfaces in *Δemb, ΔaftA, ΔubiA* and *ΔaftB*, possibly reflecting localized changes in envelope composition or organization. Young's modulus measurements (Fig. 5B) provide a more sensitive indicator of envelope disruption.

The wild-type strain had an average Young's modulus of around 180 ± 5 MPa (Fig. 5B), consistent with the robust, multilayered architecture of the corynebacterial envelope. All mutants exhibited some degree of reduced stiffness, confirming that disruptions in cell envelope biosynthesis compromise its mechanical strength.

The most dramatic decreases were observed in strains lacking components involved in arabinogalactan (AG) biosynthesis correlating with the AFM images (Fig. 5A). In particular, the *ΔaftA* mutant, which lacks an enzyme essential for initiating arabinan chain formation, showed a substantial decrease in Young's modulus of 18 ± 3 MPa, indicating a drop in stiffness. Similarly, *Δemb* (57 ± 2 MPa) and *ΔaftB* (64 ± 5 MPa), which affect later stages of arabinan polymerization and branching, also displayed a reduction in their Young's modulus and therefore, reduced stiffness (57 MPa and 64 MPa, respectively). These results highlight the importance of AG integrity in maintaining overall envelope stiffness. The Young's modulus of the *ΔubiA* mutant also showed considerable reduction (53 ± 5 MPa), indicating reduced stiffness and a broader role for membrane-associated components in supporting the integrity of the membrane and its mechanical properties. In contrast, the *Δpks13* mutant, lacking a key enzyme in mycolic acid biosynthesis, showed a less pronounced reduction (85 ± 15 MPa), indicating that although mycolic acids contribute to the cell wall's mechanical profile, they are not as central to stiffness as AG.

These findings underscore the critical role of arabinogalactan as a structural scaffold in the corynebacterial envelope. The progressive loss of stiffness across AG-deficient mutants supports the idea that AG is essential for preserving the envelope's biophysical integrity.

## 4 Discussion

The cell envelope of *C. glutamicum* is a complex, multilayered structure whose integrity depends on the precise coordination between its key components, particularly the peptidoglycan-arabinogalactan-mycolic acid framework. Our comprehensive analysis of mutants lacking essential biosynthetic genes highlights the intricate interplay between these layers and reveals how perturbations in one component causes a disturbance through the entire envelope, producing diverse biochemical, physical, and functional effects.

### 4.1 Interdependence of envelope layers shapes structure and function

Central to the envelope's architecture is the dynamic relationship between the arabinogalactan (AG)-peptidoglycan scaffold and the outer mycolic acid layer. Our lipid profiling (Fig. 3) shows that mutations affecting arabinogalactan biosynthesis or modification (e.g., Δ*aftA*, Δ*emb*, Δ*aftB*, Δ*ubiA*) not only disrupt the polysaccharide backbone but also impair the proper incorporation and transport of mycolic acid-containing glycolipids like TMCM and TDCM. The resulting accumulation of these glycolipids likely reflects a block in their integration due to defects in the underlying AG layer. The complete absence of mycolic acids in the Δ*pks13* mutant eliminates these glycolipids entirely and leads to the buildup of alternative lipid species, indicating that defects in the outer lipid layer influence intracellular lipid metabolism as well. This bidirectional communication between layers underscores that the envelope functions as a highly integrated system.

### 4.2 Cell surface hydrophobicity depends on layer integration

The hexadecane partitioning assay (Fig. 4) further illustrates this layered interdependence. Mycolic acids primarily determine surface hydrophobicity, a critical trait for environmental interactions and antibiotic exclusion. However, the degree of hydrophobicity loss correlates with how much arabinogalactan disruption affects mycolic acid attachment and retention. For instance, the Δ*aftB* mutant, which retains some mycolic acids, shows a less severe hydrophobicity decrease than Δ*emb* or Δ*aftA* mutants with more pronounced envelope defects. This suggests that the presentation and functionality of mycolic acids are tightly linked to the structural integrity of the underlying polysaccharide scaffold.

### 4.3 Envelope integrity governs antibiotic susceptibility and metabolic adaptation

The increased sensitivity to rifampicin observed in mutants with altered mycolic acid content further emphasizes the envelope's role as a permeability barrier (Table 2). This barrier depends not just on the presence of mycolic acids but also on their correct incorporation into the cell wall, which in turn requires a functional arabinogalactan scaffold. The accumulation of FAMEs and novel lipid species in certain mutants suggests the existence of metabolic feedback loops that adjust lipid synthesis in response to envelope assembly defects.

These compensatory mechanisms point to a tightly regulated envelope biogenesis process where disruption in one biosynthetic module triggers adjustments elsewhere, offering potential new targets for antimicrobial strategies that simultaneously disrupt multiple envelope layers.

### 4.4 Mechanical stability is anchored by arabinogalactan but modulated by mycolic acids

Atomic force microscopy reveals that envelope rigidity largely depends on the arabinogalactan and peptidoglycan matrix (Fig. 5). Mutants with defects in arabinogalactan biosynthesis exhibit dramatic drops in stiffness, emphasizing the polysaccharide scaffold's central mechanical role. These strains also showed a higher sensitivity to antibiotics and lower hydrophobicity suggesting the role of the AG-peptidoglycan layer in preserving the barrier function of the membrane along with its mechanical integrity. The Δ*pks13* mutant, despite lacking mycolic acids, shows a comparatively smaller stiffness reduction, implying that mycolic acids fine-tune envelope flexibility rather than provide primary structural support. This division of labor reflects the envelope's sophisticated design: the polysaccharide backbone provides mechanical strength and shape, while the mycolic acid layer contributes biochemical properties and modulates overall stability. Disruption of the AG layer thus compromises both the envelope's physical robustness and its ability to properly display mycolic acids, highlighting the delicate balance maintained through interlayer cooperation.

### 4.5 Implications for pathogenic bacteria

Insights into how envelope architecture determines rigidity, permeability, and hydrophobicity have broader significance for pathogenic actinobacteria, such as *M. tuberculosis* and *M. abscessus*, where the complex cell wall underpins intrinsic drug resistance and biofilm-mediated persistence (Dokic et al., 2021; Chakraborty et al., 2021). The interdependence between cell wall layers observed in *C. glutamicum* suggests that disruption of one biosynthetic pathway, such as arabinogalactan assembly, could potentiate the effects of inhibitors targeting mycolic acid synthesis or transport. This mechanistic understanding opens opportunities for the rational design of combination therapies that exploit multiple points of vulnerability within the envelope. The effects of loss of cell wall integrity identified may help explain how pathogenic mycobacteria regulate biofilm development and environmental resilience, providing new directions for interventions aimed at preventing surface adhesion and persistent infection.

## 5 Conclusion

In summary, our data reveal the *C. glutamicum* cell envelope as a highly interconnected and interdependent system. Disturbances in one layer propagate through the structure, affecting biochemical properties, mechanical strength, and drug susceptibility. Recognizing this complexity is essential for designing effective therapies that exploit multiple vulnerabilities within the envelope and for developing diagnostic tools that detect unique biophysical signatures of envelope defects. This integrated perspective on cell envelope architecture not only enhances our understanding of actinobacterial biology but also provides a foundation for identifying combination drug targets that exploit the interdependence of arabinogalactan and mycolic acid synthesis pathways.

## CRediT authorship contribution statement

**Mamata Modak:** Writing – review & editing, Writing – original draft, Investigation, Data curation, Conceptualization. **Uchenna Watson Waturuocha:** Writing – review & editing, Writing – original draft, Funding acquisition, Formal analysis. **Deepak Kumar Saini:** Writing – review & editing, Writing – original draft, Conceptualization. **Apoorva Bhatt:** Writing – review & editing, Writing – original draft, Funding acquisition, Conceptualization. **Gurdyal S. Besra:** Writing – review & editing, Writing – original draft, Visualization, Project administration, Funding acquisition, Conceptualization.

## Declaration of competing interest

The authors declare the following financial interests/personal relationships which may be considered as potential competing interests: Gurdyal Singh Besra and Apoorva report financial support was provided by Medical Research Council. Mamata Modak reports financial support was provided by Darwin Trust of Edinburgh. Gurdyal Singh Besra reports a relationship with the Cell Surface as an Editor.

## Data Availability

Data will be made available on request.
